# Ginsenoside 20(S)-Rg3 Targets HIF-1α to Block Hypoxia-Induced Epithelial-Mesenchymal Transition in Ovarian Cancer Cells

**DOI:** 10.1371/journal.pone.0103887

**Published:** 2014-09-08

**Authors:** Ting Liu, Le Zhao, Yan Zhang, Wei Chen, Dan Liu, Huilian Hou, Lu Ding, Xu Li

**Affiliations:** 1 Center for Translational Medicine, the First Affiliated Hospital School of Medicine, Xi'an Jiaotong University, Xi'an, China; 2 Center for Laboratory Medicine, the First Affiliated Hospital School of Medicine, Xi'an Jiaotong University, Xi'an, China; 3 Department of Gynaecology and Obstetrics, the First Affiliated Hospital School of Medicine, Xi'an Jiaotong University, Xi'an, China; 4 Department of Pathology, the First Affiliated Hospital School of Medicine, Xi'an Jiaotong University, Xi'an, China; University of Pécs Medical School, Hungary

## Abstract

The prognosis of patients with ovarian cancer has remained poor mainly because of aggressive cancer progression. Since epithelial-mesenchymal transition (EMT) is an important mechanism mediating invasion and metastasis of cancer cells, targeting the EMT process with more efficacious and less toxic compounds to inhibit metastasis is of great therapeutic value for the treatment of ovarian cancer. We have found for the first time that the ginsenoside 20(S)-Rg3, a pharmacologically active component of the traditional Chinese herb *Panax ginseng*, potently blocks hypoxia-induced EMT of ovarian cancer cells *in vitro* and *in vivo*. Mechanistic studies confirm the mode of action of 20(S)-Rg3, which reduces the expression of hypoxia-inducible factor 1α (HIF-1α) by activating the ubiquitin-proteasome pathway to promote HIF-1α degradation. A decrease in HIF-1α in turn leads to up-regulation, via transcriptional suppression of Snail, of the epithelial cell-specific marker E-cadherin and down-regulation of the mesenchymal cell-specific marker vimentin under hypoxic conditions. Importantly, 20(S)-Rg3 effectively inhibits EMT in nude mouse xenograft models of ovarian cancer, promising a novel therapeutic agent for anticancer therapy.

## Introduction

Ovarian cancer, existing predominantly in the form of epithelial ovarian cancer (EOC), is the most lethal gynecologic malignancy [1], [2]. The low survival rate of patients with ovarian cancer is associated with its vicious nature of invasion and metastasis. Among the critical contributors to the invasive and metastatic capability of ovarian cancer cells is hypoxia of the tumor microenvironment [3], [4], which mediates tumor cell invasion and metastasis through stabilization of hypoxia-inducible factor-1 alpha (HIF-1α) [5], [6].

In normoxic cells, HIF-1α is hydroxylated by a family of proline hydroxylases (PHD1-3) at Pro402 and Pro564, leading to a conformational change that promotes HIF-1α binding to the von Hippel Lindau protein (VHL) - a component of the large E3 ubiquitin ligase complex that mediates proteasomal degradation of HIF-1α [7], [8]. Under hypoxic conditions, low oxygen levels preclude proline hydroxylation, which leads to HIF-1α stabilization and subsequent formation of a heterodimer with constitutively expressed HIF-1β. The bioactive HIF-1 dimer regulates, as a transcription factor, the expression of a broad range of genes involved not only in cell cycle, apoptosis [9], angiogenesis [10], [11] and metabolism [12] in response to the hypoxic environment, but also in a cellular process called epithelial-mesenchymal transition (EMT) [13]–[15].

Identified first in embryonic development, EMT has been found later to be closely involved in the process of cancer invasion and metastasis [16], [17]. During the EMT process cells undergo a transition from a polarized epithelial phenotype to a mesenchymal phenotype [18], a biological event characterized by cell morphology change from a cobble-stone shape to a dispersed fibroblastoid shape, loss of epithelial cell-specific protein markers and acquisition of mesenchymal cell-specific properties, and enhanced cell motility and invasion. While a variety of factors including growth factors and a hypoxic microenvironment are demonstrated as inducers of EMT, many transcription factors such as Snail are confirmed as important mediators of EMT [19]. EMT suppresses epithelial cell-specific E-cadherin via action of various mediators, leading to the loss of apical-basal polarity and cell-cell adhesion junction, and up-regulates mesenchymal cell-specific vimentin [20] resulting in cytoskeleton rearrangement and cell motility enhancement. These events constitute the fundamental features of EMT at the molecular level. Of note, EMT is also implicated in anticancer drug resistance and hampers effective therapeutic intervention of cancers in advanced stages [21]. Obviously, targeting the EMT process will provide a new idea for cancer therapy, especially for ovarian cancer—a tumor that displays a strong potential to metastasize.

Ginsenosides are the pharmacologically active components of *Panax ginseng* [22] that has long been utilized as a traditional Chinese medicine for officinal or recuperative purposes [23], [24]. To date, more than 40 ginsenoside compounds have been identified [25]. Some of them, Rg1, Rg3, Rh1 and Rh2, for example, show the potent anticancer activity [24], [26]–[28]. Ginsenoside Rg3, a bioactive extracts of *Panax ginseng*, has various medical effects, such as anti-tumor [29]–[32], anti-oxidant, anti-inflammation properties [33], [34], inhibiting scar hyperplasia of skin [35] and angiogenesis [36]. In addition, stereoisomers 20(R)-Rg3 and 20(S)-Rg3 are two optically active chiral molecules differing in the orientation of the hydroxyl (OH) group on carbon-20 [24]. Although both have been reported to inhibit tumor metastasis [30], stereospecifity of Rg3 appears to be linked to their bioactivities. In this study, we have discovered for the first time that 20(S)-Rg3 effectively inhibits hypoxia-induced EMT of human ovarian cancer cells not only *in vitro* but also in nude mouse xenograft models, promising a novel natural agent for anti-ovarian cancer therapy.

## Materials and Methods

### Drugs and antibodies

20(S)-Rg3 and 20(R)-Rg3 were obtained from Tasly Pharmaceutical Company (Tianjin, China), and the purity was ≥99% as determined by high performance liquid chromatography (HPLC). Rg3 stereoisomers were dissolved in dimethyl sulfoxide (DMSO) in a 4 mg/ml stock solution and stored at −20°C. Aliquots of stock solution were added directly to the culture media.

The following antibodies were used: rabbit antibody to vimentin, VHL, mouse antibody to β-actin (Cell Signaling Technology, Beverly, MA, USA), rabbit antibody to E-cadherin (Bioworld Technology, Louis Park, MN, USA), mouse antibody to HIF-1α (Abcam Inc., Cambridge, MA, USA), sheep antibody to PHD1 (R&D Systems Inc., Minneapolis, MN, USA), rabbit antibody to Snail (Abnove, Taipei, China). Horse radish peroxidase (HRP) conjugated goat anti-mouse immunoglobulin (IgG), goat anti-mouse IgG and rabbit anti-sheep IgG (Pierce, Rockford, IL, USA), HRP-Polymer anti-Mouse/Rabbit IHC Kit (Fuzhou Maixin Biology Co. Ltd., Fouzhou, Fujian, China)

### Cell lines and culture

The human ovarian cancer cell line SKOV3 was obtained from the Shanghai Cell Bank of Chinese Academy of Sciences (Shanghai, China), 3AO was purchased from the Shandong Academy of Medical Sciences (Jinan, China).Cells were maintained in RPMI 1640 supplemented with 10% newborn bovine serum (GIBCO, Grand Island, NY, USA) under normoxic conditions (21% O_2_, 5% CO_2_, 37°C, 100% humidity) for 24 h prior to further treatment. For hypoxia induction, cells were incubated at 1% O_2_, 5% CO_2_, 94% N_2_, 37°C, 100% humidity in a HF100 hypoxia chamber (Heal Force, Hong Kong, China) for another 24 h with or without exposure to 80 µg/ml of 20(S)-Rg3 (for SKOV3) or 160 µg/ml of 20(S)-Rg3 (for 3AO), or 80,160 or 320 µg/ml of 20(R)-Rg3 (for SKOV3 and 3AO cells).

### Cell viability assay

Cells were seeded at a density of 5,000 cells per well in 96-well plates in 200 µl of RPMI 1640 containing 10% new-born bovine serum (NBS). After 24h incubation, increasing concentrations of 20(S)-Rg3 and 20(R)-Rg3 were added. 3-(4,5-dimethylthiazol-2-yl)-2,5- diphenyltetrazolium bromide (MTT) assays were undertaken after 24 h and 48 h treatment, respectively. 20 µl of 5 mg/ml MTT was added into each well. Cells were then incubated for 4 h at 37°C followed by adding of 150 µl of DMSO. The 570 nm wave-length absorption values were measured using EnSpire multimode plate reader (PerkinElmer, Waltham, MA, USA). All experiments were performed in triplicate, and repeated three times.

### Real-time polymerase chain reaction (PCR)

Total RNA was extracted from cultured cells using RNAfast 200 (Fastagen Biotech Co. Ltd., Shanghai, China) according to the manufacturer's instructions. RNA concentration and purity were determined on a UV spectrophotometer (BioRad Inc., Hercules, CA, USA) by the 260 nm absorbance and 260/280 nm absorbance ratio, respectively. cDNA synthesis was conducted using RevertAid first strand cDNA synthesis Kit (Thermo Fisher Scientific Inc., Waltham, MA, USA) according to the manufacturer's instructions. The reactions were performed at 25°C for 5 min, followed by 42°C for 60 min and 70°C for 5 min. The cDNAs were stored at −80°C for later use. Quantitative real-time PCR was performed on a CFX-96 real-time PCR system (Bio Rad, Hercules, CA, USA) using SYBR Green Master Mix (Takara Biotechnology Co. Ltd., Dalian, China). For normalization, the gene β-actin was used. Cycling conditions were as follows: initial denaturation at 95°C for 30 s, followed by 40 cycles of 95°C for 5 s and 60°C for 31 s. Each measurement was performed in triplicate, and no-template controls were included for each assay. After PCR, a dissociation curve analysis was done. Relative gene expression was calculated automatically using the 2^−ΔΔCt^ method. All oligonucleotide primers were designed and synthesized by Shanghai Shenggong Biotechnological Ltd. (Shanghai, China). The following primer sequences were used: HIF-1α-forward, 5′-ATCCATGTGACCATGAGGAAATG-3′; HIF-1α-reverse, 5′-TCGGCTAGTTAGGGTACACTTC-3′; PHD1-forward, 5′-AGGCTGTCGAAGCATTGGTG-3′; PHD1- reverse, 5′-GGGATTGTCAACGTGCCTTAC-3′; PHD2-forward, 5′-AAGCCCAGTTTGCTGACATT-3′; PHD2-reverse, 5′-TTACCGACCGAATCTGAAGG-3′; PHD3-forward, 5′-AGCCCATTTTTGCCAGACTCC-3′; PHD3-reverse, 5′-GATTTCAGAGCACGGTCAGTC-3′; VHL-forward, 5′-GCAGGCGTCGAAGAGTACG-3′; VHL- reverse, 5′-CGGACTGCGATTGCAGAAGA-3′; Snail-forward, 5′-TCCAGAGTTTACCTTCCAGCA-3′; Snail- reverse, 5′-CTTTCCCACTGTCCTCATCTG-3′; β-actin-forward, 5′- TCCCTGGAGAAGAGCTACGA-3′; β-actin- reverse, 5′-AGCACTGTGTTGGCGTACAG-3′.

### Western Blot

Total cell protein extracts were prepared by lysing cells in RIPA buffer on ice. The cells were collected by scraping and transferred to microfuge tubes. The samples were briefly sonicated and centrifuged at 12,000 rpm for 30 min to remove insoluble debris. The supernatant was collected and quantified using the Quick Start Bradford Protein Assay kit (Bio-Rad, Hercules, CA, USA). Proteins were boiled before being separated by electrophoresis on sodium dodecyl sulfate -polyacrylamide gel electrophoresis (SDS-PAGE) gels and transferred onto nitrocellulose membranes (Pall Life Science, NY, USA) using a wet transmembrane device (Amersham Pharmacia Biotech., Piscataway, NJ, USA). The membranes were blocked with 5% non-fat milk at room temperature for 1 hour, probed overnight with primary antibody (E-cadherin 1∶500, vimentin 1∶2000, β-actin 1∶1000, HIF-1α 1∶500, PHD1 1∶2000, VHL 1∶1000) in TBST followed by incubation with appropriate horse radish peroxidase (HRP) conjugated secondary antibody (goat anti-mouse/rabbit IgG 1∶2000, rabbit anti-sheep IgG 1∶1000) as indicated. ECL reagent (Santa Cruz Biotechnology, Santa Cruz, CA, USA) was used to develop the blots. All values were normalized to β-actin.

### Wound healing assay

Cells were seeded onto 6-well plates 24 h before treatment. The monolayers were scratched with a 200 µl pipette tip and washed with media without serum to remove the detached cells. Cells were maintained in media without serum under normoxia, hypoxia or hypoxia in the presence of 20(S)-Rg3 for 24 hours, respectively. The wounded areas were then imaged after incubation for an indicated period.

### Cell migration assay

Cells (1×10^5^/well) in 100 µl of media without serum were added into millicells with 8 µm pore size (Millipore Co., Bedford, MA, USA), inserted into wells containing RPMI 1640 with 20% fetal bovine serum as chemotactic factor. After 24 h incubation, the cells remaining on the upper surface of the filter were removed with a cotton swab, and the migrating cells were fixed with 5% glutaric dialdehyde followed by Giemsa staining for quantitation of cell number. The numbers of migrating cells in three high power fields of the lower surfaces of the membranes were counted. A minimum of three wells were counted per experiment.

### Small interfering RNA (siRNA) knockdown

Knockdown of PHD1 and VHL were performed using specific siRNAs purchased from GenePharma Company (Shanghai, China). Sequences of siRNAs are as follows: siPHD1-A, 5′-GCAUCACCUGUAUCUAUUATT-3′; siPHD1-B, 5′-CCAACAUCGAGCCACUCUUTT-3′; siPHD1-C, 5′-GCUAGCAUCAGGACAGAAATT-3′; siVHL-A,5′-CCACCCAAAUGUGCAGAAATT-3′; siVHL-B, 5′-GUCUCAUUCUCAGAGUAAATT-3′; siVHL-C, 5′-AACUGAAUUAUUUGUGCCAUCTT-3′. A scrambled siRNA (5′-UUCUCCGAACGUGUCACGUTT-3′) was used in parallel experiments as negative control. siRNAs were transfected to SKOV3 and 3AO cells at 40–50% confluence with siRNA transfection reagent (Roche, Indianapolis, IN, USA) according to the manufacturer's instructions. Effective siRNAs were screened out according to the results of real-time RT-PCR after 48 h incubation and by western blot after 72 h, respectively. Then the effective siRNAs were transfected into cells and incubated for 24 h followed by hypoxia induction with or without 20(S)-Rg3 treatment for another 24 h. The effect of PHD1 and VHL silence on suppression of HIF-1α by 20(S)-Rg3 was assessed at protein level.

### Luciferase reporter assay

After being seeded onto 24-well plates for 24 h, cells were transiently transfected with E-cadherin promoter luciferase reporter plasmid pGL2Basic-E-cadK1 (Addgene, Cambridge, MA, USA) and phRL-TK vector (Promega, Madison, WI, USA). 24 h later, the cells were treated with or without 20(S)-Rg3 in hypoxic settings for 24 h with normoxically cultured transfected cell as control. The cell lysate was harvested 24 h later and luciferase activity was measured by a luminometer (Promega, Madison, WI, USA) using the Dual-Luciferase Reporter System (Promega, Madison, WI, USA). E-cadherin luciferase activity was normalized to that of Renilla luciferase. Results were obtained from three independent experiments and each assay containing triplicate wells.

### Animal studies

The care and use of experimental animals were approved by the ethical committee of the First Affiliated Hospital, and were adherent to the institutional guidelines and ethical standards. All six-week old BALB/c female nude mice were housed in SPF barrier facilities under a 12 hour light/dark cycle. Animal body weights and subcutaneous tumor perpendicular diameters were recorded every two days. Tumor volumes were calculated according to the formula of V = 0.5236×(L×W^2^)(V: tumor volume, L: length, W: width).

For subcutaneous xenograft experiment, SKOV3 cells were trypsinized and resuspended in PBS at a final concentration of 2×10^7^ cells/ml. 2×10^6^ cells were injected subcutaneously into the flank of the mice. 10 days later, nude mice were randomly assigned to treatment group (n = 4) and control group (n = 4). Mice in these two group were treated by intravenous injections of either 5 mg/kg of 20(S)-Rg3 or equal volume of PBS into the tail vein every other day thereafter. After 30 days on the experimental treatments, the mice were euthanized by CO_2_ asphyxia. Tumor samples were collected and fixed in 4% paraformaldehyde for 24 h at room temperature, paraffin-embedded, and sectioned for immunohistochemical analysis.

For intraperitoneal xenograft model, SKOV3 cells were trypsinized and resuspended in PBS at a concentration of 1×10^8^/ml. Mice were inoculated with 1×10^7^ cells in the abdominal cavity at day 0. From day 1, 5 mg/kg of 20(S)-Rg3 (treatment group, n = 7) or equal volume of PBS (control group, n = 7) was injected via tail-vein every other day. The mice were observed daily and sacrificed after 30 days. Necropsies were performed, disseminated tumors were resected from mice, and ascitic fluid was collected. Total tumor weight and volume of ascitic fluid in each mouse were measured. All the contents of animal experiment we have reported here are in accordance with the ARRIVE guidelines.

### Immunohistochemistry

Histologic slides were prepared from the paraffin-embedded ovarian cancer tissue blocks. Specimens were dewaxed in xylene, rehydrated in a descending alcohol series followed by heated in 0.01 M citrate buffer (pH 6.0) in a steamer for 1.5 minutes to retrieve antigenic binding sites. Detection of antigens was carried out using incubation with the primary antibodies (E-cadherin 1∶200, vimentin 1∶400, HIF-1α 1∶200), for 2 hours at room temperature, followed by incubation with HRP-labeled secondary antibody (MaxVision HRP-Polymer anti-Mouse/Rabbit IHC Kit, 1∶200) at room temperature for 30 minutes and color development with DAB. Negative control specimens were incubated in PBS without the primary antibody under the same conditions. Slides were counterstained with hematoxylin, dehydrated in an ascending alcohol series, and mounted for analysis. Digital images were acquired on an Olympus BH-2 microscope (Tokyo, Japan) installed with the DeltaPix Camera and software (Maalov, Denmark).

### Statistical analysis

Statistical differences were determined by two-tailed *t*-test or Wilcoxon rank-sum test (only for the volume of ascites). All statistical analyses were performed using SPSS software (Chicago, IL, USA). Differences were considered significant (*) at *P*<0.05 and highly significant (**) at *P*<0.01 for all comparison.

## Results

### 20(S)-Rg3, but not 20(R)-Rg3, blocks hypoxia-induced EMT in SKOV3 and 3AO ovarian cancer cells

We first examined the effect of 20(S)-Rg3 and 20(R)-Rg3 on cell viability of the two human ovarian cancer cell lines SKOV3 and 3AO in a series of MTT assays. The structures of 20(S)-Rg3 and 20(R)-Rg3 were shown in Figure S1-A. 20(S)-Rg3 inhibited cell growth in a dose-dependent manner, giving rise to IC_50_ (inhibitory concentration at which 50% cell viability is inhibited) values of 146.8 and 242.6 µg/ml, respectively, for SKOV3 and 3AO cells 24 h (or 48 h) after treatment (Figure S1-B.). By contrast, 20(R)-Rg3 showed no apparent inhibition of the proliferation of both cell types at concentrations up to 640 µg/ml (Figure S1 A-C).

**Figure 1:**
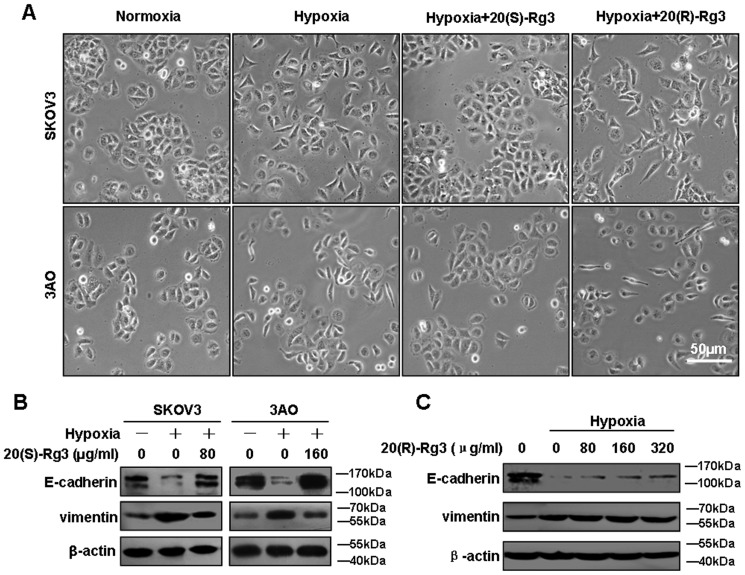
20(S)-Rg3 blocked hypoxia-induced EMT in SKOV3 and 3AO ovarian cancer cells. (A) Cells were first cultured in common condition for 24 h. For hypoxia induction in vitro, SKOV3 and 3AO cells were incubated in an hypoxia chamber of 1% O_2_, 5% CO_2_ and 94% N_2_ for another 24 h without (hypoxia) or with Rg3 (hypoxia+Rg3) at indicated concentration for 24 h. Controls were cultured under normoxic condition (normoxia) for 24 h. Cell images were captured using a phase contrast microscopy (100×). (B) Proteins of cells exposed to normoxia, hypoxia and hypoxia plus 20(S)-Rg3 were harvested, and expression of E-cadherin and vimentin were examined by western blot, using β-actin as a loading control. Hypoxia decrease E-cadherin protein and increase vimentin protein, which was reversed by 20(S)-Rg3 co-treatment. (C) The effect of 20(R)-Rg3 on expression of EMT markers in SKOV3 cells. No effect of 20(R)-Rg3 on expression of hypoxia-induced EMT markers were observed. All of the treatments in this figure were carried out in triplicate.

**Figure 2:**
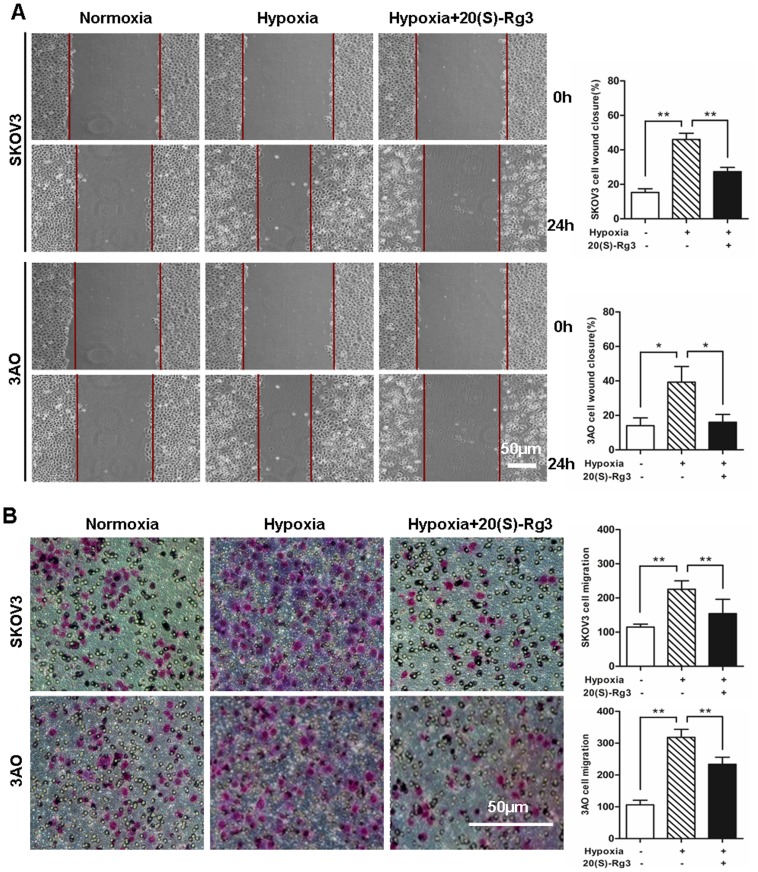
20(S)-Rg3 inhibited SKOV3 and 3AO cells mobility. (A) Cell mobility detected by wound healing assay. Cells were incubated under normoxic condition for 24 h followed by scratching the confluent cell layer and exposing to normoxia, hypoxia or hypoxia plus 20(S)-Rg3 for another 24 h, respectively. The closure of the scratch was monitored during 24 h and photographs were shown at 0 h and 24 h after scratching. (B) Cell mobility examined by *in vitro* migration assay. Normoxically cultured cells were seeded into millicells, followed by incubation for 24 h under normoxia, hypoxia or hypoxia plus 20(S)-Rg3, respectively. The number of cells that passed through the membrane per 20× magnified lens were counted and used to represent the migration capacities of cells. All of the treatments in this figure were carried out in triplicate, and the results are displayed as the means ± SD. *P<0.05, **P<0.01, *t*-test.

We next examined the ability of 20(S)-Rg3 and 20(R)-Rg3 to influence hypoxia-induced EMT. As shown in Fig. 1A, when exposed to 1% O_2_ for 24 hours both SKOV3 and 3AO cells underwent EMT characterized by a cell morphology change from the tight-junctioned cobblestone-like appearance to a dissociated spindle-like shape. This change was accompanied by a significant down-regulation of the epithelial marker E-cadherin and a marked up-regulation of the mesenchymal marker vimentin as revealed by Western blot analysis (Fig. 1B). Treatment of SKOV3 and 3AO cells with 20(S)-Rg3 at 80 µg/ml, 160 µg/ml, respectively, effectively prevented the morphological change associated with hypoxia-induced EMT (Fig. 1A), consistent with an elevated level of E-cadherin and a reduced level of vimentin under hypoxic conditions (Fig. 1B). In contrast, treatment of SKOV3 with 20(R)-Rg3 at 80 µg/ml and 160 µg/ml, respectively, failed to rescue the cells from undergoing EMT (Fig. 1A), as evidenced by unchanged levels of E-cadherin and vimentin under hypoxic culture condition (Fig. 1C). To further verify that 20(S)-Rg3 blocked hypoxia-induced EMT, cell mobility was evaluated in wound healing and *in vitro* migration assays. As shown in Fig. 2A and B, while hypoxia clearly promoted cell mobility and wound closure, these effects were largely counteracted by the treatment of SKOV3 and 3AO cells with 20(S)-Rg3.

Taken together, our findings strongly indicate that 20(S)-Rg3, but not 20(R)-Rg3, is capable of reversing hypoxia-induced EMT changes in cell morphology, cell markers, and cell mobility, underscoring its potential function as an inhibitor of hypoxia-induced EMT and EMT-mediated cancer metastasis.

### 20(S)-Rg3 reduces HIF-1α expression through promoting HIF-1α protein degradation in a PHD1/VHL/proteasome dependent manner

**Figure 3:**
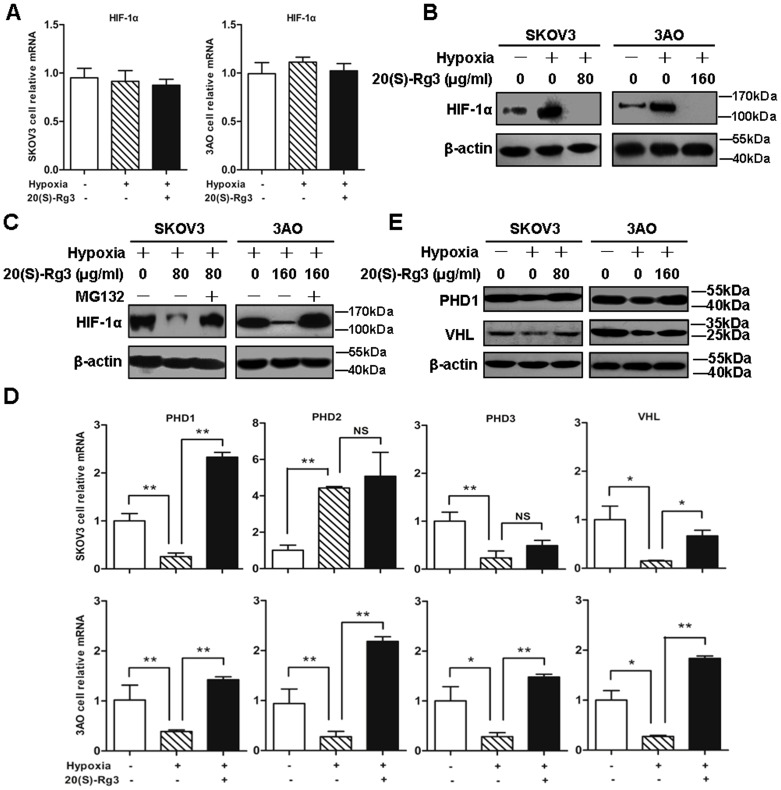
20(S)-Rg3 promoted HIF-1α degradation via PHD1-VHL-ubiquitin- proteasome pathway. (A) Total RNA from cells exposed to normoxia, hypoxia or hypoxia plus 20(S)-Rg3, respectively, for 24 h were extracted. Real-time RT-PCR assays were performed to analyze effect of 20(S)-Rg3 on HIF-1α mRNA level, and no changes were detected. (B) Cells cultured for 24 h under normoxia, hypoxia or hypoxia plus 20(S)-Rg3, respectively, were monitored by western blot for their expression of HIF-1α. 20(S)-Rg3 interfered with hypoxia-induced HIF-1α augmentation. (C) MG132 blocked the inhibition effect of 20(S)-Rg3 on HIF-1α protein level. HIF-1α protein levels were compared among hypoxic cells, hypoxic cells co-treated with 20(S)-Rg3, and cells pretreated with 20 µM MG132 for 30 mins followed by exposure to hypoxia and 20(S)-Rg3 for 24 h. (D) Cells exposed to normoxia, hypoxia and hypoxia in the presence of 20(S)-Rg3, respectively, were assessed by real-time PCR for transcription of PHD1, PHD2, PHD3 and VHL. After 20(S)-Rg3 treatment, the transcription of PHD1 and VHL genes, which were decreased under hypoxia, were increased in both SKOV3 and 3AO cells. (E) Protein extracts from treated cells were examined by western blot for expression of PHD1 and VHL, with protein from normoxically cultured cells as control and β-actin as loading control. PHD1 and VHL declined in hypoxic cells. 20(S)-Rg3 recovered their expression under hypoxia condition. All of the treatments in this figure were carried out in triplicate, and the results are displayed as the means ± SD. *P<0.05, **P<0.01, *t*-test.

To better understand the molecular mechanisms by which 20(S)-Rg3 blocks hypoxia-induced EMT in the ovarian cancer cells, we analyzed the expression of HIF-1α, a crucial regulator of EMT, at both the transcriptional and translational levels. When cultured in the presence of 1% O_2_ for 24 hours HIF-1α protein level was elevated in SKOV3 and 3AO cells, while HIF-1α mRNA level was hardly affected. Treatment of the cells with 20(S)-Rg3 in otherwise identical hypoxic settings markedly suppressed HIF-1α expression at the protein level with the mRNA level unchanged, indicating that HIF-1α was post-transcriptionally regulated by 20(S)-Rg3 (Fig. 3A and B).

Since HIF-1α protein reduction was closely related with protein degradation via the ubiquitin-proteasome pathway, the 26S proteasome-specific protease inhibitor MG132 was used to investigate whether 20(S)-Rg3 promoted proteasomal degradation of HIF-1α. As shown in Fig. 3C, pretreatment of 20 µM of MG132 for 30 min under hypoxic conditions restored HIF-1α protein in SKOV3 and 3AO cells treated with 20(S)-Rg3, demonstrating that 20(S)-Rg3 down-regulated HIF-1α in the proteasome-dependent manner. As members of the PHD family and the VHL protein are known factors mediating ubiquitin/proteasome-dependent HIF-1α degradation, the effect of 20(S)-Rg3 on the mRNA levels of PHD1, PHD2, PHD3 and VHL was further examined. Real-time PCR data showed that hypoxia consistently down-regulated PHD1, PHD3 and VHL (Fig. 3D). This effect of hypoxia, especially on PHD1 and VHL, however, was reversed by 20(S)-Rg3 in both SKOV3 and 3AO cells. As expected, PHD1 and VHL protein levels were largely restored, as was the case for mRNA, following the treatment of SKOV3 and 3AO cells with 20(S)-Rg3 to counteract hypoxia-mediated down-regulation of PHD1 and VHL proteins (Fig. 3E).

**Figure 4:**
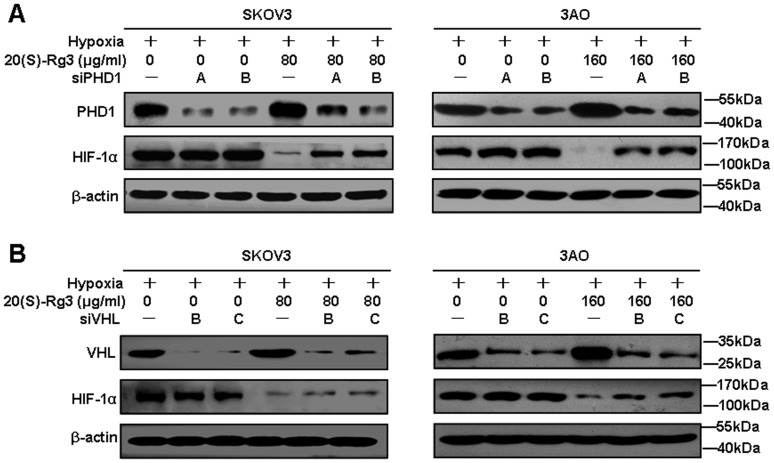
Deficiency of PHD1 and VHL counteracted inhibitory effect of 20(S)-Rg3 on HIF-1α protein level. Effect of PHD1(A) and VHL (B)silence on 20(S)-Rg3-suppressed HIF-1α. SKOV3 and 3AO cells were transiently transfected for 24 h with siPHD1 or siVHL, followed by incubation under hypoxic conditions for another 24 h. The protein level of PHD1, VHL and HIF-1α were then detected using western blot. All experiments were repeated at least three times.

To further elucidate the roles of PHD1 and VHL in mediating the activity of 20(S)-Rg3 with respect to HIF-1α degradation, SKOV3 and 3AO cells were transduced with siRNA-PHD1 (siPHD1) or siRNA-VHL (siVHL). Relying on real-time PCR and western blot, we selected two most effective siRNA for silencing PHD1 (siPHD1-A and siPHD1-B) and VHL (siVHL-B and siVHL-C) respectively (Figure S2), followed by hypoxia stimulation and 20(S)-Rg3 treatment. As determined by western blot, knock-down of PHD1 or VHL inhibited the pro-degradation effect 20(S)-Rg3 on HIF-1α (Fig. 4A and B), with the effect of siPHD1 more significant than that of siVHL on HIF-1α protein recovery. Collectively, these results suggest that 20(S)-Rg3 blocked hypoxia-induced EMT of the ovarian cancer cells by activating the PHD1- VHL- ubiquitin/proteasome pathway to facilitate HIF-1α degradation.

### 20(S)-Rg3 abolishes hypoxia-induced transcriptional repression of E-cadherin through suppressing Snail

**Figure 5:**
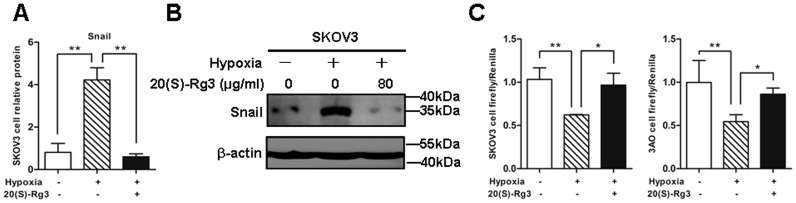
Effects of 20(S)-Rg3 on Snail expression and E-cadherin promoter activity. (A) SKOV3 cells were cultured in normal condition for 24 h, and then cultured in 21% O_2_ (normoxia) or 1% O_2_ (hypoxia) or 1% O_2_ and 80 µg/ml 20(S)-Rg3 (hypoxia+20(S)-Rg3) for another 24 h. Snail mRNA was determined by RT-PCR with β-actin as an inner control, and standardized against the level present in normoxically cultured cells. The expression of Snail was increased at mRNA level in SKOV3 cells after hypoxia stimulation for 24 h. 20(S)-Rg3 treatment abrogated Snail upregulation caused by hypoxia. (B) Cell extracts were subjected to immunoblot analysis to detect Snail protein level, and β-actin was used as a loading control. Low level of Snail protein was detected in normoxically cultured cells, while Snail protein was increased in hypoxic cells. 20(S)-Rg3 reduced hypoxia-induced Snail expression. (C) Luciferase assay in SKOV3 and 3AO cells. E-cadherin promoter activity was significantly reduced in hypoxic cells. Decreases in E-cadherin promoter activity were reversed by 20(S)-Rg3 co-treatment. Activity of the E-cadherin promoter firefly luciferase constructs was normalized to that of a cotransfected *Renilla* luciferase construct. All of the treatments in this figure were carried out in triplicate, and values are presented as the means ± SD of three experiments. *P<0.05, **P<0.01, for *t*-test.

During EMT process, E-cadherin is commonly suppressed by its transcriptional repressors including Snail which itself is transcriptionally activated by HIF-1. To better understand how 20(S)-Rg3 prevented hypoxia-induced down-regulation of E-cadherin, we investigated the effect of 20(S)-Rg3 on Snail. Reverse- transcription PCR results showed that hypoxia elicited a drastic increase of Snail mRNA, which apparently was inhibited by 20(S)-Rg3 (Fig. 5A). Consequently, the up-regulation of Snail protein stimulated by hypoxia was severely attenuated by 20(S)-Rg3 treatment (Fig. 5B).

To further investigate whether repression of Snail by 20(S)-Rg3 was responsible for the recovery of E-cadherin under hypoxic conditions, a dual-luciferase reporter assay was carried out. An E-cadherin promoter-luciferase reporter construct and an internal control vector capable of expressing Renilla luciferase were co-transfected into cells. Hypoxia was found to render almost 50% loss of the luciferase signal intensity in both SKOV3 and 3AO cells, while 20(S)-Rg3 suppressed hypoxic effects and caused a significant increase in luciferase intensity (Fig. 5C). These results demonstrated that 20(S)-Rg3 blocked hypoxic inhibition of E-cadherin promoter activity.

### 20(S)-Rg3 inhibits ovarian cancer growth and EMT *in vivo*

**Figure 6:**
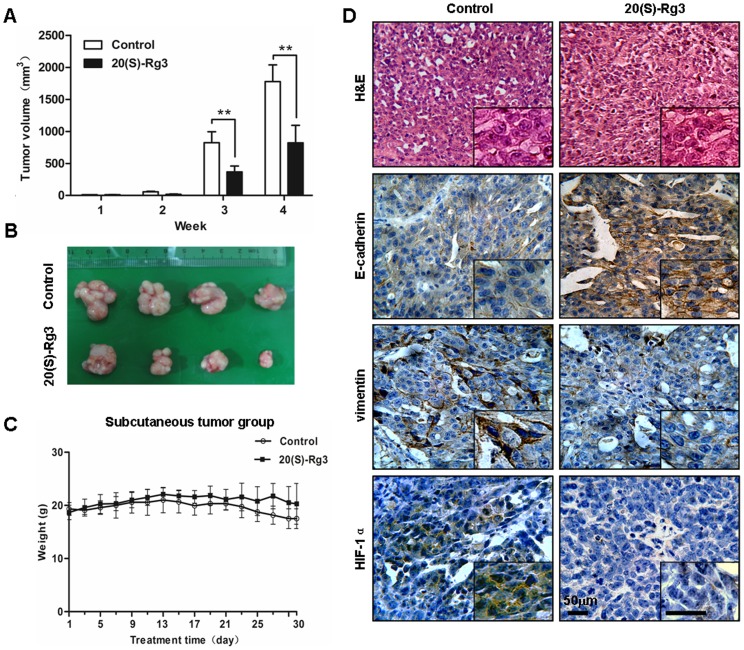
20(S)-Rg3 inhibits ovarian cancer growth and EMT in nude mice. (A) SKOV3 cells were injected subcutaneously into the nude mice flank 2 weeks before 20(S)-Rg3 was intravenously injected into a tail vein every other day at a dose of 5 mg/kg for 30 days. (A) The volume of primary tumors were calculated after the treatment of PBS or 20(S)-Rg3 for 4 weeks. 20(S)-Rg3 strongly inhibited tumor growth in nude mice. Data represents the mean (mm^3^) ± SD (n = 4 per groups). (B) Images of the primary tumors from PBS-treated or 20(S)-Rg3-treated mice. (C) The body weights of nude mice with subcutaneous xenograft were monitored every other day. No differences in body weight were observed between 20(S)-Rg3-treated and PBS-treated mice. Values are presented as the means (g) ± SD (n = 4 per groups). (D) Immunohistochemical staining of E-cadherin, vimentin and expression in subcutaneous tumor samples (original magnification, 200×; insets, 400×). 20(S)-Rg3 enhanced E-cadherin but weakened vimentin and HIF-1α *in vivo.* *P<0.05, **P<0.01, for *t*-test.

To determine the effect of 20(S)-Rg3 on tumor progression *in vivo*, the primary tumor growth in nude mice were examined. Animals were given either 20(S)-Rg3 or PBS by intravenous injection every other day beginning 10 days after subcutaneous inoculation of 2×10^6^ cells into the flank and the primary tumor sizes were monitored every week. While all mice developed tumors, 20(S)-Rg3 treatment led to a nearly 50% reduction in tumor volume at week 4 (Fig. 6A, B) compared with the PBS-treated controls. While there was no difference in body weight between PBS treated and 20(S)-Rg3 treated mice (Fig. 6C). To further investigate whether 20(S)-Rg3 could transform the mesenchymal traits of SKOV3 cells into epithelial phenotypes *in vivo*, the subcutaneous tumors were fixated for confirmation by H&E staining and immunohistochemistry analysis of E-cadherin, vimentin and HIF-1α. As shown in Fig. 6D, subcutaneous tumors in control mice exhibited a high level of vimentin and HIF-1α, and a low level of E-cadherin. Contrarily, tumors in 20(S)-Rg3 treated mice displayed E-cadherin elevation, and vimentin and HIF-1α decrease. These *in vivo* findings were consistent with the *in vitro* changes observed in the hypoxia-induced EMT cell models, demonstrating that 20(S)-Rg3 robustly blocked EMT process in ovarian cancer.

**Figure 7:**
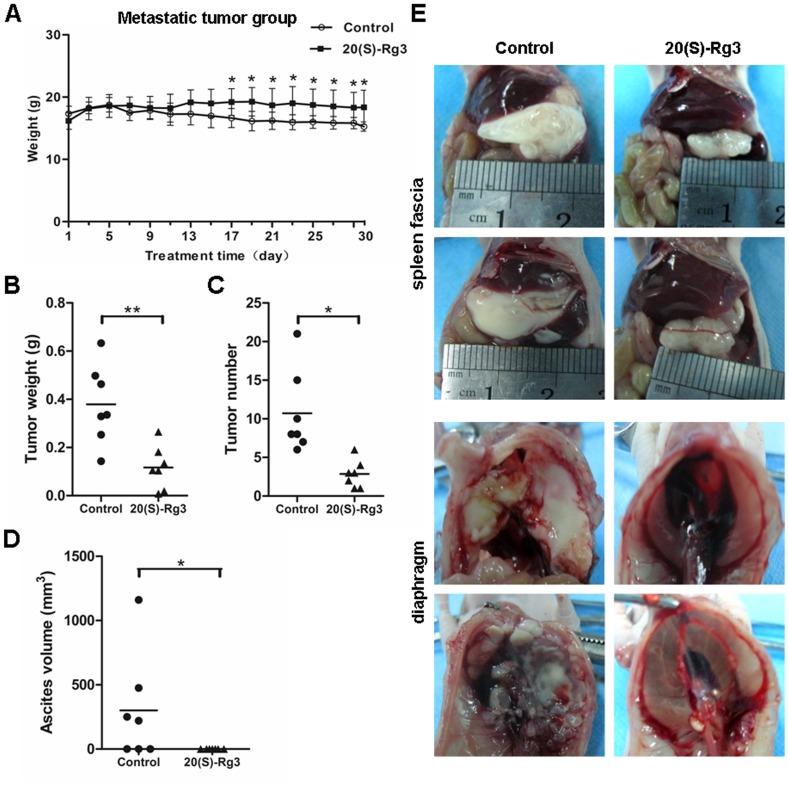
20(S)-Rg3 suppressed ovarian cancer intraperitoneal dissemination in vivo. Mice injected i.p. with SKOV3 cells underwent 20(S)-Rg3 treatment for 30 days. (A) The body weights of nude mice with metastatic tumors were monitored every other day. Mice in 20(S)-Rg3 group lost less body weight than those given PBS. The overall tumor weight (B), the number of metastases (C) and the volume of ascites (D) were measured. Data are presented as the means (g) ± SD (n = 7 per groups). *P<0.05, **P<0.01, for *t*-test(picture A, B and C) and Wilcoxon test (picture D), respectively. (E) The representative images of xenografts of spleen fascia and diaphragm. Those xenografts in 20(S)-Rg3-treated group were obviously smaller in size than those in control group.

Having shown that 20(S)-Rg3 possessed significant activity against hypoxia-induced EMT and tumor growth *in vivo*, we next examined the inhibitory effect of 20(S)-Rg3 on the intraperitoneal spread of ovarian cancer produced by SKOV3 cells in nude mice. Mice intravenously administered with 20(S)-Rg3 at a dosage of 5 mg/kg every other day after tumor cell inoculation into the abdomen. Compared with the control group, 20(S)-Rg3 decreased the total weight of tumors (Fig. 7B), the number of metastatic tumor nodules (Fig. 7C) and the volume of ascites (Fig. 7D) after 30-day treatment. In addition, the body weights of 20(S)-Rg3-treated mice were higher than that of controlled mice (Fig. 7A). Intraperitoneal tumor nodules grew throughout the mice's abdominal cavity. It's worth noting that xenografts of spleen fascia and diaphragm of treated mice were substantially smaller in size than those in untreated animals(Fig. 7E). These datas suggest that 20(S)-Rg3 could suppress dissemination of ovarian cancer cell in abdominal cavity.

## Discussion

Ovarian cancer, a highly lethal malignancy of gynecological tumor, often spread through the abdominal cavity and metastasize to distant organs in advanced stages. EMT plays a critical role in ovarian cancer progression, because it can promote ability of migration, invasion, drug resistance of ovarian cancer [37]. Targeting EMT in ovarian cancer thus provides an attractive therapeutic method [38]. The hypoxic tumor microenvironment is an amportant factor in the induction of a tumor EMT, which facilitates invasion and distant metastasis of ovarian cancer. Hypoxia as part of the pro-metastatic niche realizes its function mainly through stabilization of HIF-1α [39]. Growing evidence demonstrates that hypoxia and HIF-1α are valid anti-cancer drug targets [39], [40].Therapeutic compounds specific for hypoxia and HIF-1α, and particularly those with less cytotoxicity, will likely enjoy clinical benefits not shared by the commonly used genotoxic chemo drugs that indiscriminately kill both normal and cancer cells and result in severe side effects and treatment failure. In this regard, searching pharmacologically active ingredients from natural sources such as Chinese herbs to inhibit tumor growth and metastasis by modulating the hypoxic tumor microenvironment represents an attractive therapeutic approach to anticancer drug discovery. In this study, we have demonstrated for the first time that the ginsenoside 20(S)-Rg3, isolated from the traditional Chinese herb *Panax ginseng*, effectively inhibits ovarian cancer migration and intraperitoneal dissemination by inducing HIF-1α degradation and blocking hypoxia-driven EMT of cancer cells.

20(S)-Rg3 has a broad spectrum of anti-tumor activities, ranging from the prevention of tumor development and the inhibition of tumor progression to the enhancement of chemotherapeutic response, among others. The mechanisms of action of 20(S)-Rg3 vary, however. It has been reported that 20(S)-Rg3 can prevent 12-*O*-tetradecanoylphorbol-13-acetate (TPA)-induced skin tumor growth partly through NF-κB inactivation-mediated reduction of COX-2 and suppression of DNA binding activity of AP-1 [34]. 20(S)-Rg3 has also been shown to restrain HT29 colorectal cancer cell proliferation by inhibiting mitosis and inducing apoptosis [29]. While 20(S)-Rg3 promotes TRAIL-induced apoptosis in hepatocellular carcinoma cells via C/EBP homology protein-mediated DR5 upregulation [41], it decimates lung metastasis produced by B16-BL6 melanoma in syngeneic mice through inhibiting angiogenesis and reducing cancer cell adhesion and invasion [30]. More recently, Kim et al. demonstrated that 20(S)-Rg3 sensitized prostate cancer cells to docetaxel and other chemotherapeutics by inhibiting cell growth and augmenting apoptosis via suppression of constitutively activated and TNFα-induced NF-κB [42]. These anti-tumor activities of 20(S)-Rg3 against diverse cancers underscore its therapeutic potential as a broad-spectrum anti-cancer drug molecule.

The published work clearly indicates that the mechanisms by which 20(S)-Rg3 inhibits tumor growth and metastasis *in vitro* and *in vivo* are cell type dependent. This observation has motivated us to study the effect of 20(S)-Rg3 on EMT-induced ovarian cancer progression, which has not previously been reported. In our model system, 20(S)-Rg3 abrogated hypoxia-induced EMT *in vitro* by reducing HIF-1α at the protein level. This reduction was achieved via enhanced degradation of HIF-1α protein dependent on the pathways composed of PHD1, VHL and proteasome. HIF-1α degradation seemed dependent more on the activity of proteasome than on PHD1 and VHL, since proteasome inhibitor MG132 could completely block 20(S)-Rg3 inhibition of HIF-1α under hypoxic conditions. Importantly, 20(S)-Rg3, at low doses, exerted similarly effective inhibitory activity *in vivo* against tumor intraperitoneal spread via reducing HIF-1α protein level and reversing EMT process. Of note, Zhang and colleagues have recently shown that ginsenoside 25-OCH_3_-PPD, isolated from *Panax notoginseng* and belonging to protopanaxadiols group as 20(S)-Rg3, is capable of reducing EMT markers in normoxically cultured breast cancer cells [43].Xie et al. demonstrated that ginsenoside Rg1, a major active component also isolated from *Panax notoginseng* but belonging to protopanaxatriol group, blocked TGFβ1-induced EMT in rat renal tubular epithelial cells [44].

Collectively, these findings, together with our *in vitro* and *in vivo* data, shed new light on the anti-EMT mechanism of ginsenosides in general, and validate potential clinical use of 20(S)-Rg3 in particular as adjuvant chemotherapeutics for patients with advanced ovarian cancer.

The therapeutic use as a new class of anticancer drugs of pharmacologically active natural compounds present in Chinese herbs, and particularly those isolated from medically proven and clinically safe ginseng products, would be obviously advantageous compared with the more conventional genotoxic chemo drugs. 20(S)-Rg3 is non-cytotoxic to normal cells [41], which it more attractive than most HIF-1α inhibitors under therapeutic development. However, hurdles exist to the eventual translation of 20(S)-Rg3 to clinical use. One of them entails the lack of knowledge still about the molecular target(s) 20(S)-Rg3 directly interacts with to induce the various biological effects seen in this study. This paucity of 20(S)-Rg3 targets hinders the elucidation of precise upstream signaling events leading to the inhibition of hypoxia-induced EMT, and poses technical challenges to structure-based function improvement of 20(S)-Rg3 for even better therapeutic efficacy *in vivo*. Moreover, since 20(S)-Rg3 is active against a variety of human cancers [29], [30], [45], it remains to be seen whether or not this ginsenoside also blocks EMT in other well-characterized tumor models. Our work reported here lays ground for necessary additional studies of this existing compound to warrant its clinical use as a new anti-cancer drug for the treatment of various metastatic diseases including ovarian cancer.

## Supporting Information

Figure S1 **The anti-proliferation activity of Rg3 to ovarian cancer cells.** (A) Structure of 20(S)-Rg3 and 20(R)-Rg3. (B) Growth inhibition effect of 20(S)-Rg3 on SKOV3 and 3AO cells after 24 h and 48 h treatment. (C) Effect of 20(R)-Rg3 on SKOV3 and 3AO cell growth after 24 h and 48 h treatment. All of the treatments in this figure were carried out in triplicate, and values are presented as the means ± SD of three experiments. *P<0.05, **P<0.01, for *t*-test.
(TIF)

Figure S2 **Screening of effective siRNAs to PHD1 and VHL.** SKOV3 cells were transfected for 48 h with siPHD1, siVHL or scrambled control siRNA, and mRNA and protein levels were determined. Left panel: screening of effective siPHD1 and siVHL by real-time PCR. Right panel: screening of effective siPHD1 and siVHL by western blot. All of the treatments in this figure were carried out in triplicate, and values are presented as the means ± SD of three experiments. *P<0.05, **P<0.01, for *t*-test.
(TIF)
