# Supplementary figures and images for: Ginsenoside 20(S)-Rg3 Targets HIF-1α to Block Hypoxia-Induced Epithelial-Mesenchymal Transition in Ovarian Cancer Cells
Source: PLoS One. 2014 Sep 8;9(9):e103887. doi: 10.1371/journal.pone.0103887 (PMC4157750; doi:10.1371/journal.pone.0103887)

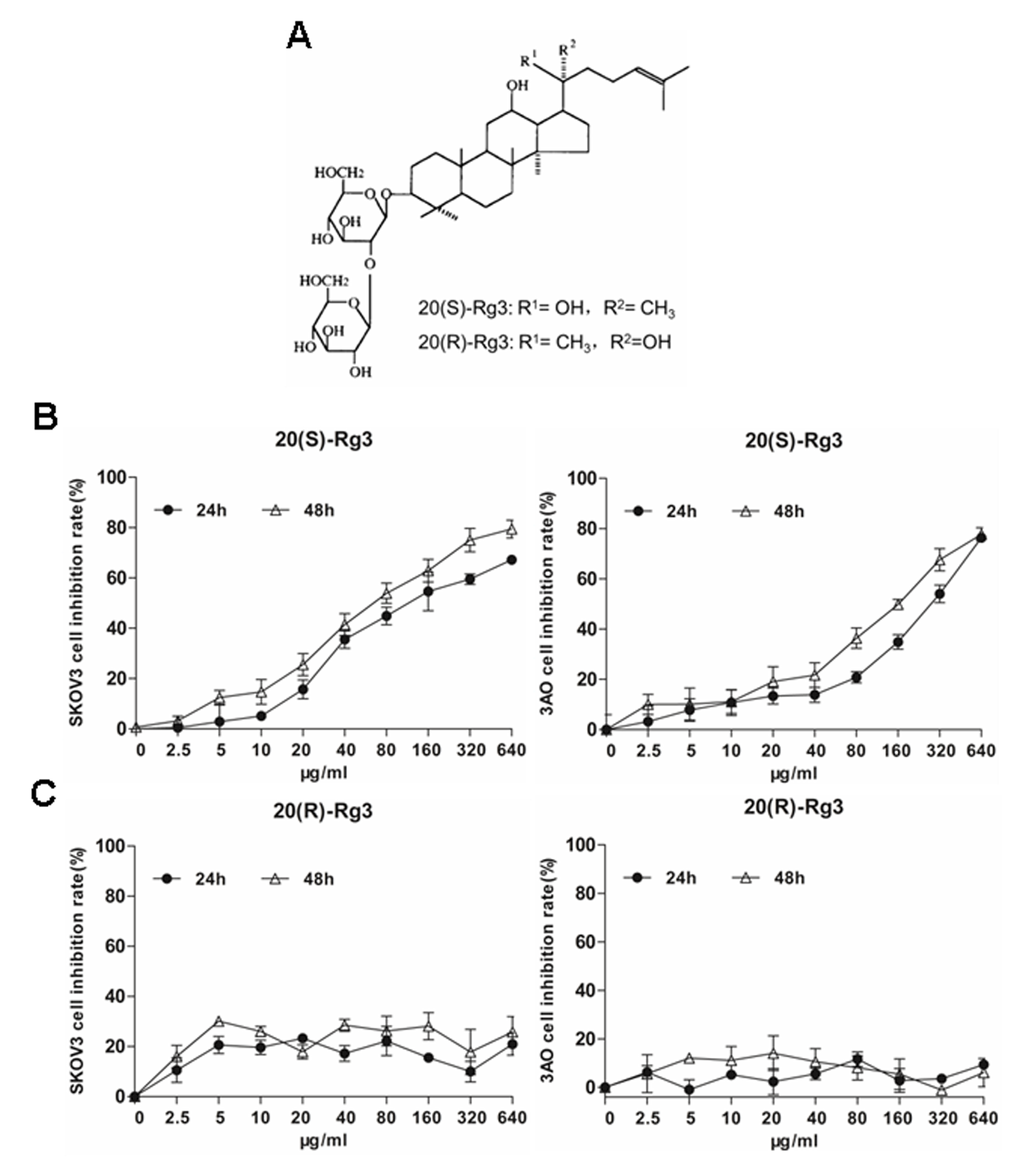

Supplement: Figure S1 — The anti-proliferation activity of Rg3 to ovarian cancer cells. (A) Structure of 20(S)-Rg3 and 20(R)-Rg3. (B) Growth inhibition effect of 20(S)-Rg3 on SKOV3 and 3AO cells after 24 h and 48 h treatment. (C) Effect of 20(R)-Rg3 on SKOV3 and 3AO cell growth after 24 h and 48 h treatment. All of the treatments in this figure were carried out in triplicate, and values are presented as the means ± SD of three experiments. *P<0.05, **P<0.01, for t-test. (TIF) [file pone.0103887.s001.tif]

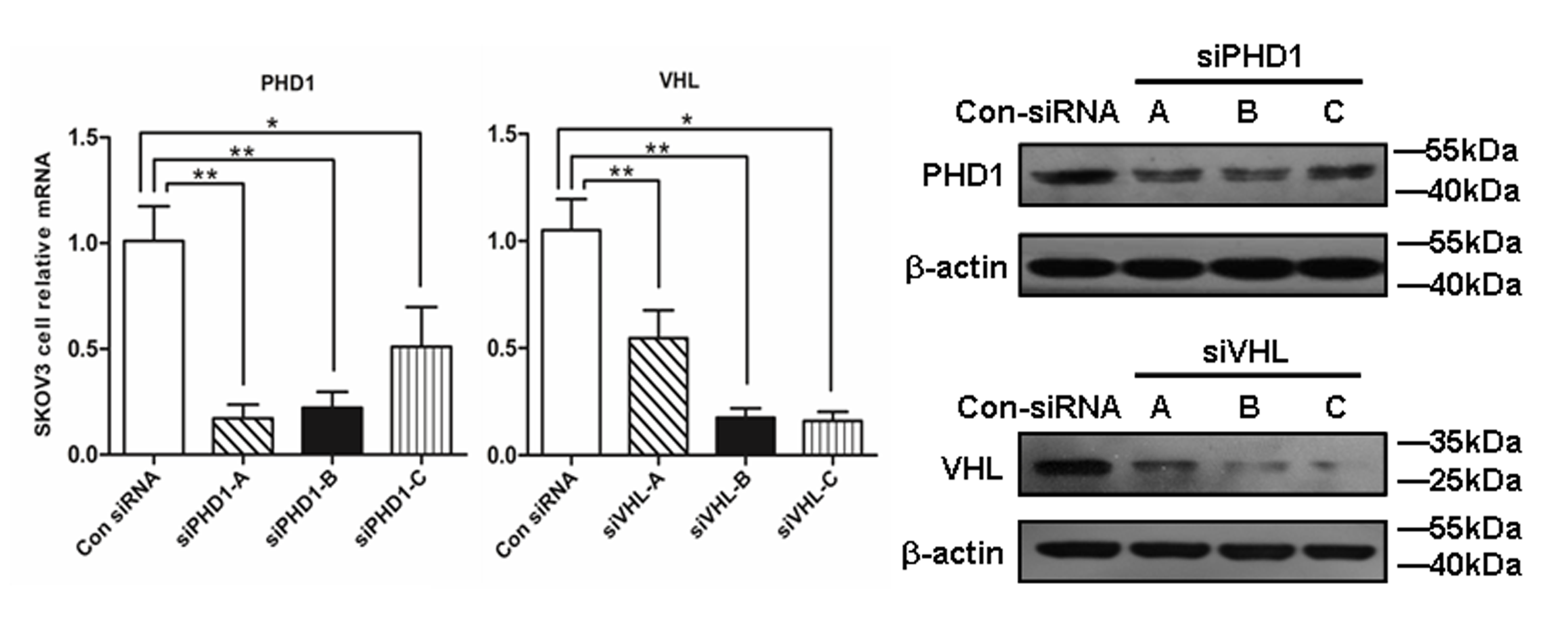

Supplement: Figure S2 — Screening of effective siRNAs to PHD1 and VHL. SKOV3 cells were transfected for 48 h with siPHD1, siVHL or scrambled control siRNA, and mRNA and protein levels were determined. Left panel: screening of effective siPHD1 and siVHL by real-time PCR. Right panel: screening of effective siPHD1 and siVHL by western blot. All of the treatments in this figure were carried out in triplicate, and values are presented as the means ± SD of three experiments. *P<0.05, **P<0.01, for t-test. (TIF) [file pone.0103887.s002.tif]
